# Impact of low versus negative estrogen/progesterone receptor status on clinico-pathologic characteristics and survival outcomes in HER2-negative breast cancer

**DOI:** 10.1038/s41523-022-00448-4

**Published:** 2022-07-11

**Authors:** Rachel Yoder, Bruce F. Kimler, Joshua M. Staley, Kelsey Schwensen, Yen Y. Wang, Karissa Finke, Anne O’Dea, Lauren Nye, Manana Elia, Gregory Crane, Richard McKittrick, Robert Pluenneke, Sheshadri Madhusudhana, Larry Beck, Anuj Shrestha, Larry Corum, Mark Marsico, Shane R. Stecklein, Andrew K. Godwin, Qamar J. Khan, Priyanka Sharma

**Affiliations:** 1 University of Kansas Cancer Center, Kansas City, KS USA; 2 Department of Radiation Oncology, University of Kansas Medical Center, Kansas City, KS USA; 3 Department of Internal Medicine, University of Kansas Medical Center, Westwood, KS USA; 4 Clinical Trials Shared Resource, University of Kansas Medical Center, Westwood, KS USA; 5 Department of Internal Medicine, University of Kansas Medical Center, Lee’s Summit, MO USA; 6 Department of Internal Medicine, University of Kansas Medical Center, Overland Park, KS USA; 7 Department of Internal Medicine, University of Kansas Medical Center, Kansas City, MO USA; 8 Department of Internal Medicine, University of Missouri-Kansas City, Kansas City, MO USA; 9 Tammy Walker Cancer Center, Salina Regional Health Center, Salina, KS USA; 10 Richard & Annette Bloch Cancer Center, Truman Medical Center, Kansas City, MO USA; 11 Olathe Cancer Care, Olathe Medical Center, Olathe, KS USA; 12 Department of Pharmacoepidemiology/Oncology, Merck & Co., Inc, Kenilworth, NJ USA; 13 Department of Pathology & Laboratory Medicine, University of Kansas Medical Center, Kansas City, KS USA

**Keywords:** Breast cancer, Tumour biomarkers, Epidemiology

## Abstract

Triple-negative breast cancer (TNBC) is classically defined by estrogen receptor (ER) and progesterone receptor (PR) immunohistochemistry expression <1% and absence of *HER2* amplification/overexpression. HER2-negative breast cancer with low ER/PR expression (1–10%) has a gene expression profile similar to TNBC; however, real-world treatment patterns, chemotherapy response, endocrine therapy benefit, and survival outcomes for the Low-ER group are not well known. 516 patients with stage I-III HER2-negative breast cancer and ER/PR expression ≤10% who were enrolled in a multisite prospective registry between 2011 and 2019 were categorized on the basis of ER/PR expression. TNBC (ER and PR < 1%) and Low-ER (ER and/or PR 1–10%) groups comprised 87.4% (*n* = 451) and 12.6% (*n* = 65) of patients, respectively. Demographic, clinical, and treatment characteristics, including prevalence of germline *BRCA1/2* mutation, racial and ethnic distribution, and chemotherapy use were not different between TNBC and Low-ER groups. No difference was observed in recurrence-free survival (RFS) and overall survival (OS) between TNBC and Low-ER groups (3-year RFS 82.5% versus 82.4%, respectively, *p* = 0.728; 3-year OS 88.0% versus 83.4%, respectively, *p* = 0.632). Among 358 patients receiving neoadjuvant chemotherapy, rates of pathologic complete response were similar for TNBC and Low-ER groups (49.2% vs 51.3%, respectively, *p* = 0.808). The HER2-negative Low-ER group is often excluded from TNBC clinical trials assessing novel treatments (immunotherapy and antibody-drug conjugates), thus limiting efficacy data for newer effective therapies in this group. Given that HER2-negative Low-ER disease displays clinical characteristics and outcomes similar to TNBC, inclusion of this group in TNBC clinical trials is encouraged.

## Introduction

Triple-negative breast cancer (TNBC), which is defined by the lack of expression of estrogen receptor (ER) and progesterone receptor (PR) and absence of *HER2* overexpression and/or gene amplification, accounts for 15% of all breast cancers in the United States. The 2010 American Society of Clinical Oncology/College of American Pathologists (ASCO/CAP) guideline recommended that invasive breast cancers with immunohistochemistry ER expression of ≥1% be considered ER-positive^1^. The primary purpose for this recommended threshold for ER positivity was to serve as a predictive marker for benefit from endocrine therapy, as some studies had suggested potential adjuvant endocrine therapy benefit in patients with as little as 1% ER expression^2^. Following the publication of these guidelines, an immunohistochemical expression of <1% for the definition of ER/PR negativity has been adopted for TNBC designation (in addition to ASCO/CAP criteria for HER2 negativity).

It has, however, been noted that HER2-negative breast cancers with low ER expression of 1–10% (Low-ER) show molecular features similar to ER-negative breast cancers and are more likely to be basal-like compared to ER-high (>10%) breast cancers^3,4^. Furthermore, there is paucity of data regarding the extent of adjuvant endocrine therapy benefit in the setting of low ER expression 1–10%. Accordingly, the 2020 ASCO/CAP update introduced a new reporting category of “ER-low positive” for breast cancers with 1–10% ER positivity, with a comment stating, “there are limited data on the overall benefit of endocrine therapies for patients with low level (1–10%) ER expression, but they currently suggest possible benefit, so patients are considered eligible for endocrine treatment”^5^. In addition, the 2020 ASCO guideline also acknowledges that “there are data that suggest invasive cancers with these results are heterogeneous in both behavior and biology and often have gene expression profiles more similar to ER-negative cancers.” Similarly, the 2015 St. Gallen Consensus reported that ER expression values between 1% and 9% should be considered equivocal and that endocrine treatment alone, in the absence of chemotherapy, should not be considered a reliable adjuvant treatment for these patients^6^. As reflected in these practice guidelines, there continues to be ambiguity regarding the clinically relevant ER threshold for endocrine therapy benefit. As a result, most clinical trials in TNBC generally exclude patients with Low-ER expression.

TNBC is associated with inferior long-term outcomes compared to other breast cancer subtypes^7,8^. Until recently, chemotherapy had remained the only available systemic treatment for patients with TNBC. However, recent years have seen advances in treatment for TNBC, with newer agents like immune checkpoint inhibitors and antibody-drug conjugates (ADCs) showing promise. In the last two years therapeutic agents have received approval from the U.S. Food and Drug Administration and the European Medicines Agency specifically for treatment of TNBC: pembrolizumab (anti-PD-1 antibody) plus chemotherapy for locally recurrent unresectable or metastatic PD-L1 positive (combined positive score ≥10) TNBC^9^; sacituzumab govitecan (an ADC) for patients with unresectable/locally advanced or metastatic TNBC who have received two or more prior systemic therapies^10^; and pembrolizumab in combination with chemotherapy as neoadjuvant treatment, and then continued as adjuvant treatment for high-risk, early-stage TNBC^11^. Despite contemporary multi-agent chemotherapy regimens, though, 20–40% of patients with early-stage TNBC develop metastatic disease, and median survival with chemotherapy alone in metastatic TNBC is under two years, emphasizing the critical need for patients with TNBC to have access to the newly available life-prolonging TNBC-specific therapies^9,10,12,13^. Supposing that HER2-negative, Low-ER breast cancer and TNBC demonstrate similar biology and clinical behavior, it is plausible that patients with Low-ER breast cancer may derive similar benefit from TNBC-specific treatments.

The current study utilizes a prospective multisite registry of patients treated in the contemporary era to assess the impact of low versus negative ER/PR expression on clinico-pathologic characteristics, treatment patterns, and survival outcomes in patients with HER2-negative breast cancers.

## Results

### Study population

Among 516 participants, median age was 53 years, 19.6% were African American, 34.3% had lymph node-positive disease, and 13.6% had germline *BRCA1/2* mutation. TNBC and Low-ER groups comprised 87.4% (451/516) and 12.6% (65/516) of patients, respectively. In the Low-ER group, ER expression ranged 1%–10% and PR expression ranged 1–3%. Within the Low-ER group, 21.5% (14/65) had both ER and PR expression 1–10%, 52.3% (34/65) had ER expression 1–10% with PR expression <1%, and 26.2% (17/65) had PR expression 1–10% with ER expression <1%. Demographic, clinico-pathologic, and treatment characteristics were balanced between TNBC and Low-ER groups (Table 1). Racial and ethnic distribution was not different between the Low-ER and TNBC groups, with similar proportion of patients with African American race in the two ER/PR expression groups.

**Table 1:** Patient characteristics.

Characteristic – *N* (%)	All	TNBC	Low-ER	*p*
	*N* = 516	*N* = 451	*N* = 65	
Age at diagnosis, years – median (range)	53 (23–97)	54 (23–97)	51 (28–76)	0.172
Race^a^				0.693
White	386 (74.8%)	335 (74.3%)	51 (78.5%)	
African American	101 (19.6%)	89 (19.7%)	12 (18.5%)	
Asian	8 (1.6%)	8 (1.8%)	0 (0%)	
Other	20 (3.9%)	18 (4.0%)	2 (3.1%)	
Ethnicity^b^				0.537
Non-Hispanic	497 (96.3%)	434 (96.2%)	63 (96.9%)	
Hispanic	14 (2.7%)	13 (2.9%)	1 (1.5%)	
Menopausal status^c^				0.127
Premenopausal	214 (41.5%)	181 (40.1%)	33 (50.8%)	
Postmenopausal	295 (57.2%)	263 (58.3%)	32 (49.2%)	
TNM stage				0.102
Stage I	179 (34.7%)	150 (33.3%)	29 (44.6%)	
Stage II	263 (51.0%)	232 (51.4%)	31 (47.7%)	
Stage III	74 (14.3%)	69 (15.3%)	5 (7.7%)	
T stage				0.256
TX	3 (0.6%)	3 (0.7%)	0 (0.0%)	
T1	183 (35.7%)	153 (34.2%)	30 (46.2%)	
T2	263 (51.3%)	235 (52.5%)	28 (43.1%)	
T3	56 (10.9%)	49 (10.9%)	7 (10.8%)	
T4	11 (2.1%)	11 (2.5%)	0 (0.0%)	
Nodal status				0.357
Positive	117 (34.3%)	158 (35.0%)	19 (29.2%)	
Negative	339 (65.7%)	293 (65.0%)	46 (70.8%)	
Histological grade				0.824
I	2 (0.4%)	2 (0.4%)	0 (0.0%)	
II	86 (16.7%)	76 (16.9%)	10 (15.4%)	
III	428 (82.9%)	373 (82.7%)	55 (84.6%)	
Germline *BRCA1/2* deleterious mutation				0.530
Yes	70 (13.6%)	64 (14.2%)	6 (9.2%)	
No	357 (69.2%)	309 (68.5%)	48 (73.8%)	
Unknown	89 (17.2%)	78 (17.3%)	11 (16.9%)	
Chemotherapy received				0.307
Neoadjuvant only	300 (58.1%)	269 (59.6%)	31 (47.7%)	
Adjuvant only	146 (28.3%)	122 (27.1%)	24 (36.9%)	
Neoadjuvant + adjuvant	58 (11.2%)	50 (11.1%)	8 (12.3%)	
None	12 (2.3%)	10 (2.2%)	2 (3.1%)	
Neo/adjuvant chemotherapy regimen	*N* = 504	*N* = 441	*N* = 63	0.269
Anthracycline-taxane-based	138 (27.4%)	123 (27.9%)	15 (23.8%)	
Anthracycline-taxane-based plus platinum	91 (18.1%)	81 (18.4%)	10 (15.9%)	
Taxane-based	61 (12.1%)	51 (11.6%)	10 (15.9%)	
Taxane-platinum-based	185 (36.7%)	164 (37.2%)	21 (33.3%)	
Other	29 (5.8%)	22 (5.0%)	7 (11.1%)	
pCR (in patients with neoadjuvant chemotherapy)	*N* = 358	*N* = 319	*N* = 39	0.808
Yes	177 (49.4%)	157 (49.2%)	20 (51.3%)	
No	181 (50.6%)	162 (50.8%)	19 (48.7%)	
Surgery type^d^				0.227
Lumpectomy	205 (39.8%)	173 (38.4%)	32 (49.2%)	
Mastectomy	308 (59.8%)	275 (61.1%)	33 (50.8%)	
None	2 (0.4%)	2 (0.4%)	0 (0.0%)	
Adjuvant radiation therapy^e^				0.978
Yes	304 (59.8%)	265 (59.8%)	39 (60.0%)	
No	204 (40.2%)	178 (40.2%)	26 (40.0%)	
Adjuvant endocrine therapy				<0.001
Yes	20 (3.9%)	7 (1.6%)	13 (20.0%)	
No	496 (96.1%)	444 (98.4%)	52 (80.0%)	

^a^Race unknown for *n* = 1 patient.

^b^Ethnicity unknown for *n* = 5 patients.

^c^Menopausal status unknown for *n* = 7 patients.

^d^Surgery type unknown for *n* = 1 patient.

^e^Radiation therapy status unknown for *n* = 8 patients.

A numerically higher proportion of patients in the Low-ER group had stage I disease compared to those in the TNBC group, although this difference was not statistically significant (44.6% vs 33.3%, *p* = 0.102). Similarly, a numerically higher proportion of patients in the Low-ER group had node-negative disease compared to those in the TNBC group (70.8% vs 65.0%, *p* = 0.357). Overall, 97.7% of patients received systemic chemotherapy (97.8% in Low-ER and 96.9% in TNBC). The types of chemotherapy regimen used in the Low-ER and TNBC groups were similar. Compared to the TNBC group, a numerically lower proportion of patients in the Low-ER group received neoadjuvant vs adjuvant chemotherapy (60.0% vs 70.7%, *p* = 0.079); the numerical differences in neoadjuvant chemotherapy use are likely due to somewhat lower presentation stage in the Low-ER compared to TNBC group. Among patients receiving neoadjuvant chemotherapy, rates of pCR were similar in TNBC and Low-ER groups. Pathologic complete response was observed in 49.2% and 51.3% of patients in TNBC and Low-ER groups, respectively (*p* = 0.808) (Table 1). Twenty percent (13/65) of patients in the Low-ER group and 1.6% (7/451) in the TNBC group received adjuvant endocrine therapy (*p* < 0.001). No demographic, clinico-pathologic, or treatment characteristics were found to correlate with receipt of endocrine therapy within the Low-ER group, though number of patients in these analyses was small.

### Survival outcomes

**Fig. 1:**
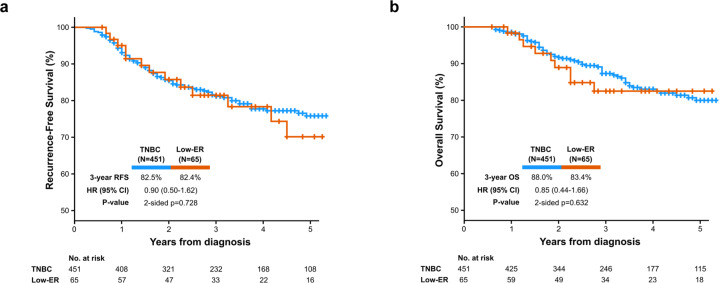
Survival by ER/PR expression group. **a** Recurrence-free survival. **b** Overall survival.

**Fig. 2:**
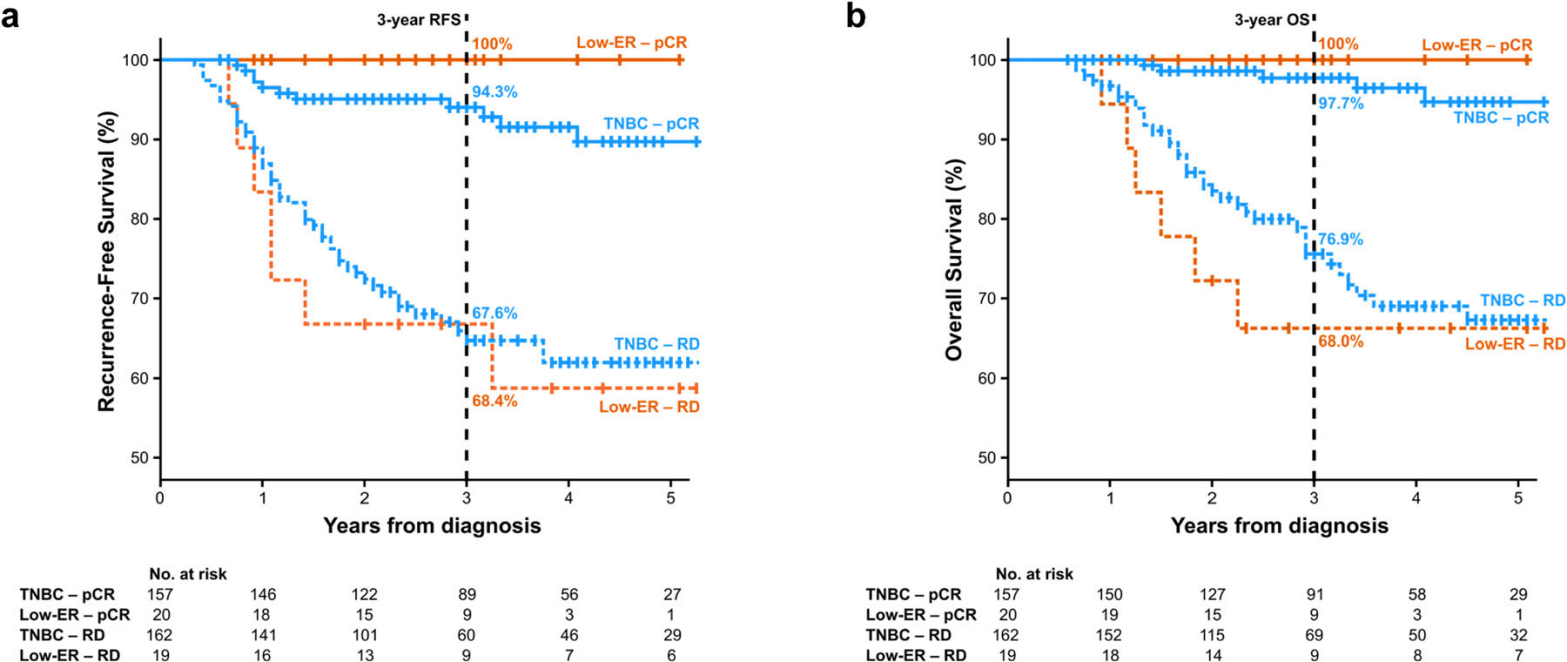
Survival by pathologic response and ER/PR expression group. **a** Recurrence-free survival. **b** Overall survival. RD residual disease.

At a median follow-up of 39 months (range: 7–124 months), there have been 82 recurrences (distant *N* = 66, local/regional *N* = 15, site of metastasis unknown N = 1) and 72 deaths. There were 69 recurrences in the TNBC group (distant N = 57, local/regional *N* = 11, site of metastasis unknown *N* = 1) and 13 in the Low-ER group (distant *N* = 9, local/regional *N* = 4). There was no significant difference in RFS and OS between the TNBC and Low-ER groups. Three-year RFS was 82.5% and 82.4% among TNBC and Low-ER groups, respectively (hazard ratio (HR) 0.90; 95% CI: 0.50–1.62, *p* = 0.728) (Fig. 1a). Three-year OS was 88.0% and 83.4% among TNBC and Low-ER groups, respectively (HR 0.85; 95% CI: 0.44–1.66, *p* = 0.632) (Fig. 1b). Among patients receiving neoadjuvant chemotherapy, those achieving pCR demonstrated significantly better RFS and OS compared to patients without pCR. Estimated 3-year RFS was 95.0% and 67.8% in patients with and without pCR, respectively (HR 0.17; 95% CI: 0.09–0.33, *p* < 0.001). Estimated 3-year OS was 97.9% and 75.9% for patients with and without pCR, respectively (HR 0.13; 95% CI: 0.06–0.30, *p* < 0.001). For patients who received neoadjuvant chemotherapy, survival outcomes were also assessed by pCR status and ER/PR expression. Among patients with residual disease, RFS was similar in the TNBC and Low-ER groups (3-year RFS 67.6% in TNBC and 68.4% in Low-ER, HR 0.88, 95% CI: 0.40–1.93, *p* = 0.738), with similar observations for OS (3-year OS 76.9% in TNBC and 68.0% in Low-ER, HR 0.77, 95% CI: 0.32–1.81, *p* = 0.540) (Fig. 2). Among patients achieving pCR, 3-year RFS was also similar in the TNBC and Low-ER groups (3-year RFS 94.3% in TNBC and 100% in Low-ER), with similar observations for OS (3-year OS 97.7% in TNBC and 100% in Low-ER). There were no EFS or OS events among the 20 patients with pCR in the Low-ER group, prohibiting log-rank comparison.

On univariable analysis, nodal positivity, higher T stage, and receipt of radiation therapy were significantly associated with inferior RFS (HR 2.65, *p* < 0.001; HR 3.42, *p* < 0.001; and HR 1.77, *p* = 0.011, respectively) and OS (HR 3.28, *p* < 0.001; HR 4.40, p < 0.001; and HR 1.85, *p* = 0.018, respectively), and mastectomy (compared to lumpectomy) was associated with trend towards inferior RFS (HR 1.53, p = 0.053) and OS (HR 1.56, *p* = 0.082) (Table 2). On multivariable analysis (included variables: nodal status, T stage, radiation therapy, ER/PR expression, type of surgery, endocrine therapy), node positivity and higher T stage retained significant association with inferior RFS (HR 1.77, *p* = 0.015 and HR 2.36, *p* = 0.001, respectively) and OS (HR 2.24, *p* = 0.004 and HR 3.21, *p* < 0.001, respectively).

**Table 2:** Univariable and multivariable analysis.

Univariable					
Variable		RFS		OS	
		HR (95% CI)	*p*	HR (95% CI)	*p*
Age (continuous)		1.01 (1.00–1.03)	0.125	1.02 (1.00–1.03)	0.140
Race	White	1		1	
	Non-white	1.08 (0.67–1.72)	0.754	0.94 (0.53–1.66)	0.821
Germline *BRCA1/2* mutation	Yes	1		1	
	No	1.34 (0.69–2.61)	0.383	1.67 (0.72–3.88)	0.232
ER/PR expression group	TNBC	1		1	
	Low-ER	1.11 (0.62–1.99)	0.729	1.12 (0.60–2.30)	0.633
Grade	1–2	1		1	
	3	1.15 (0.66–1.99)	0.630	0.95 (0.52–1.73)	0.865
T stage	T1/2	1		1	
	T3/4	3.42 (2.19–5.34)	<0.001	4.40 (2.69–7.19)	<0.001
Nodal status	Negative	1		1	
	Positive	2.65 (1.77–3.96)	<0.001	3.28 (2.04–5.26)	<0.001
Surgery type	Lumpectomy	1		1	
	Mastectomy	1.53 (0.99–2.37)	0.053	1.56 (0.94–2.59)	0.082
Adjuvant radiation therapy	No	1		1	
	Yes	1.77 (1.13–2.77)	0.011	1.85 (1.10–3.11)	0.018
Chemotherapy setting	Neoadjuvant	1		1	
	Adjuvant	0.81 (0.51–1.27)	0.354	0.85 (0.51–1.43)	0.547
Adjuvant endocrine therapy	No	1		1	
	Yes	1.81 (0.79–4.13)	0.155	1.56 (0.57–4.28)	0.382

Multivariable					
Variable		RFS		OS	
		HR (95% CI)	*p*	HR (95% CI)	*p*
T stage	T1/2	1		1	
	T3/4	2.36 (1.44–3.88)	0.001	3.19 (1.84–5.52)	<0.001
Nodal status	Negative	1		1	
	Positive	1.79 (1.12–2.87)	0.015	2.25 (1.31–3.89)	0.004
Surgery type	Lumpectomy	1		1	
	Mastectomy	1.39 (0.83–2.31)	0.210	1.16 (0.64–2.11)	0.615
Adjuvant radiation therapy	No	1		1	
	Yes	1.66 (0.98–2.82)	0.059	1.57 (0.85–2.89)	0.146
Adjuvant endocrine therapy	No	1		1	
	Yes	1.23 (0.48–3.17)	0.670	0.92 (0.29–2.89)	0.880
ER/PR expression group	TNBC	1		1	
	Low-ER	1.23 (0.63–2.40)	0.540	1.44 (0.67–3.06)	0.350

Within the Low-ER group, use of endocrine therapy was not associated with survival outcomes. 3-year RFS was 75.5% and 84.4% with and without endocrine therapy, respectively (HR 1.81; 95% CI: 0.56–5.90, *p* = 0.315) and 3-year OS 83.3% and 83.6% with and without endocrine therapy, respectively (HR 1.13; 95% CI: 0.24–5.38, *p* = 0.874) (Supplementary Fig. 2).

## Discussion

In this multisite real-world study, patients with HER2-negative Low-ER breast cancer comprised 12.6% of the cohort, representing a small but appreciable subset. Low-ER cancers and TNBC demonstrated similar clinico-pathologic characteristics and median age at diagnosis. Compared to TNBC, ER-positive breast cancer is less prevalent in African American women^14^; in our study, however, African American race distribution was similar between Low-ER and TNBC. Furthermore, while germline *BRCA1/2* mutations are observed at a lower frequency in ER-positive breast cancer than in TNBC^15^, the mutation prevalence among Low-ER patients in our study was not significantly different from that of the TNBC group. These rates are also in line with previous reports of a 10–15% mutation rate in unselected TNBC^16,17^. Taken together, these observations highlight key etiological similarities between TNBC and Low-ER HER2-negative breast cancer.

Chemotherapy use patterns were generally similar between the Low-ER and TNBC groups, with 97% of patients in both groups having been prescribed neoadjuvant or adjuvant chemotherapy. The high rate of chemotherapy perhaps indicates inclination of providers to manage chemotherapy recommendations for Low-ER disease similarly to those for TNBC rather than for ER-positive breast cancer, where chemotherapy is recommended less frequently. It is well known that complete pathological response is less frequent in ER-positive breast cancer compared to TNBC; in our study, pCR rates with contemporary neoadjuvant chemotherapy regimens were similar in Low-ER and TNBC patients, a finding that is consistent with previous reports^18–21^. Previous studies and meta-analyses have demonstrated that pCR is a robust surrogate for improved long-term outcomes^7,20^. Accordingly, we found achievement of pCR to be highly prognostic for RFS and OS regardless of ER expression group (Low-ER vs TNBC). Residual disease after neoadjuvant chemotherapy is associated with poor outcomes with higher hazard rates for recurrence and lower absolute survival in TNBC compared to hormone receptor-positive breast cancer^20,22^. Outcomes of patients with residual disease in our study were poor in both Low-ER and TNBC groups, with similar RFS and OS for Low-ER and TNBC in the setting of residual disease. Overall, there was no difference in RFS and OS between Low-ER breast cancer and TNBC.

Our findings are in line with other studies, which have likewise shown comparable pathological response rates and survival for Low-ER breast cancer and TNBC^18,23–26^. In our cohort, only nodal positivity and higher T stage (and not ER/PR expression group) were significantly associated with RFS and OS on multivariable analysis, consistent with previous work^24^. Taken together, these findings underscore the substantial similarities between TNBC and Low-ER, HER2-negative breast cancer with regard to clinical behavior, treatment response, and long-term outcomes.

Patients with ER/PR expression 1–10% in our cohort were treated with adjuvant endocrine therapy at a low rate of 20%. Other studies have described similar low usage of endocrine therapy among patients with Low-ER breast cancer^23,24^. Notably, as opposed to other studies, all patients in our cohort were treated after the 2010 ASCO/CAP guideline update which defined the cutoff for ER positivity as 1% expression by immunohistochemistry; however, adjuvant endocrine therapy use was still modest in the Low-ER group despite being classified as “ER-positive” per the 2010 guidelines. Consistent with other reports, endocrine therapy use did not impact survival outcomes in the Low-ER group of our study^18,25–27^. Survival analysis by endocrine therapy use in our study was limited by small numbers of patients, and these findings should be confirmed in other larger studies.

Endocrine therapy provides substantial benefit to patients with ER-positive breast cancer but is not devoid of side effects^28^. The collective lack of substantiative evidence of benefit in Low-ER disease, coupled with the known negative impact of endocrine therapy on quality of life likely contributed to low rates of adjuvant endocrine therapy use in our study as well as previous studies. The 2020 ASCO/CAP-designated “ER low positive” reporting category thus highlights the need for more definitive answers to the question of optimal ER expression threshold as it relates to endocrine therapy benefit. As a result of this uncertainty, patients with Low-ER HER2-negative breast cancer are commonly excluded from clinical trials available for patients with TNBC despite their shared clinico-pathologic characteristics, chemosensitivity, and prognosis^9,11,29^. However, some recent TNBC trials have begun expanding inclusion criteria to 5% or 10% ER/PR expression (e.g., NCT01982448, NCT02445391, NCT03639948). It is possible that significant heterogeneity exists within the subset of HER2-negative tumors expressing 1–10% ER/PR and that further biomarker analysis may uncover additional factors predictive of response to endocrine therapy and novel agents like immunotherapy and antibody drug conjugates.

Our study does have limitations, including the relatively small number of patients in the Low-ER group and lack of central testing for ER/PR. The Low-ER subgroup comprised 12.6% of our study population; this finding is consistent with previous studies which have shown that this group accounts for 8–16% of HER2-negative ER/PR-negative breast cancers, with different definitions of ER/PR negativity being utilized across studies^18,23,24^. Although lack of central ER/PR testing may be considered a limitation, utilization of local testing is representative of the real-world setting. We acknowledge that survival analysis by adjuvant therapy use in our study is based on small numbers of patients and events and should be considered hypothesis-generating. Strengths of our study include its multisite prospective nature, contemporary enrollment period (2011–2019) where patients received modern treatment, inclusion of a substantial number of patients treated with neoadjuvant chemotherapy, and availability of germline *BRCA1/2* mutation data.

In summary, breast cancers with ER/PR expression 1–10% represent a small but appreciable subset of early-stage HER2-negative disease and mimic the clinical behavior, chemosensitivity, and survival outcomes of TNBC. Our study findings add further evidence for the need for systematic evaluation of this subgroup with respect to effectiveness of novel TNBC-specific therapies and degree of potential benefit from endocrine therapy. These findings also bring to the forefront the potential need to reevaluate the ER/PR cutoff for clinical trials in TNBC. If the Low-ER group is indeed similar to TNBC, then the current approach of adhering to the 1% cutoff may lead to denying this group access to effective TNBC therapy just to ensure that some marginal endocrine therapy benefit is not lost. Findings from the current study support consideration for inclusion of patients with Low-ER HER2-negative disease in future TNBC clinical trials.

## Methods

### Study design and participants

Participants were males and females aged 18 years or older with stage I–III HER2-negative breast cancer and estrogen receptor (ER) and progesterone receptor (PR) immunohistochemical nuclear staining ≤10% who enrolled in a multisite prospective registry protocol (NCT02302742) between March 2011 and April 2019 at 14 locations/centers. HER2 negativity was determined per ASCO/CAP guidelines^1,30^.

This study was conducted in accordance with the U.S. Common Rule and the International Ethical Guidelines for Biomedical Research Involving Human Subjects. Human investigations were performed after institutional review board approval at the University of Kansas Medical Center, and all subjects provided written informed consent.

Demographic, clinical, pathologic, and treatment information (systemic and local treatment was determined by treating physicians) was collected, and participants were prospectively followed for recurrence and survival. Patients were categorized into two groups on the basis of ER and PR immunohistochemical expression (local testing). TNBC was defined as ER and PR expression <1%; Low-ER was defined as ER and/or PR expression 1–10%. Pathologic response was ascertained for patients who had undergone neoadjuvant chemotherapy; pathologic complete response (pCR) was defined as the absence of residual invasive disease in breast and axilla, with or without ductal carcinoma in situ (ypTis/T0N0).

### Statistical analysis

Baseline characteristics and pathologic response were compared across groups by chi-squared analysis, with Mann–Whitney U test being used for continuous variables. Recurrence-free survival (RFS) and overall survival (OS) were estimated according to the Kaplan–Meier method and compared across groups by log-rank test, followed by Cox regression analysis. RFS was defined as time from diagnosis to first recurrence (invasive ipsilateral breast, invasive local/regional, or distant), or to death as a result of any cause^31^. OS was defined as time from diagnosis to death as a result of any cause. Patients were censored on the date of last contact if an event had not been observed. Cox regression modeling was used for univariable and multivariable analysis of factors associated with recurrence and death. All reported p-values and confidence intervals (CI) are from two-sided tests. *P*-values <0.05 were considered statistically significant. All analyses were conducted using SPSS Statistics version 27 (IBM Corporation).

## Supplementary information

Supplementary Information

## Data Availability

The datasets generated and analyzed during the current study are not publicly available due to them containing information that could compromise research participant privacy. Additionally, explicit consent to deposit participant-level data was not obtained from participants, and many participants are deceased or lost to follow-up, which precludes obtaining consent for the data deposition. However, a limited set of de-identified data can be made available by the corresponding author (P.S.) upon reasonable request.
